# Using scenario‐based assessments to examine the feasibility of integrating preventive nutrition services through the primary health care system in Bangladesh

**DOI:** 10.1111/mcn.13366

**Published:** 2022-05-04

**Authors:** Phuong H. Nguyen, Priyanjana Pramanik, Sk. Masum Billah, Rasmi Avula, Tarana Ferdous, Bidhan K. Sarker, Musfikur Rahman, Santhia Ireen, Zeba Mahmud, Purnima Menon, Deborah Ash

**Affiliations:** ^1^ International Food Policy Research Institute Washington District of Columbia USA; ^2^ International Food Policy Research Institute New Delhi India; ^3^ International Centre for Diarrheal Disease Research, Bangladesh (icddr, b) Dhaka Bangladesh; ^4^ The University of Sydney School of Public Health Sydney New South Wales Australia; ^5^ FHI Solutions Dhaka Bangladesh

**Keywords:** antenatal care, Bangladesh, child health, community health, maternal health, preventive and promotive nutrition services, well‐child services

## Abstract

The National Nutrition Services of Bangladesh aims to deliver nutrition services through the primary health care system. Little is known about the feasibility of reshaping service delivery to close gaps in nutrition intervention coverage and utilization. We used a scenario‐based feasibility testing approach to assess potential implementation improvements to strengthen service delivery. We conducted in‐depth interviews with 31 service providers and 12 policymakers, and 5 focus group discussions with potential beneficiaries. We asked about the feasibility of four hypothetical scenarios for preventive and promotive nutrition service delivery: community‐based events (CBE) for pregnant women, well‐child services integrated into immunization contacts; CBE for well‐children, and well‐child visits at facilities. Opinions on service delivery platforms were mixed; some recommended new platforms, but others suggested strengthening existing delivery points. CBE for pregnant women was perceived as feasible, but workforce shortages emerged as a key barrier. Challenges such as equipment portability, upset children and a fast‐moving service environment suggested low feasibility of integrating nutrition into outreach immunization contacts. In contrast, CBE and facility‐based well‐child visits emerged as feasible options, conditional on having the necessary workforce, structural readiness and budget support. On the demand side, enabling factors include using interpersonal communication and involving community leaders to increase awareness, organizing events at a convenient time and place for both providers and beneficiaries, and incentives for beneficiaries to encourage participation. In conclusion, integrating preventive and promotive nutrition services require addressing current challenges in the health system, including human resource and logistic gaps, and investing in creating demand for preventive services.

## Key messages

- This study uses a scenario‐based feasibility testing approach to explore potential interventions to strengthen preventive and promotive nutrition service delivery through the primary health care system.
- Our findings highlight three highly feasible potential platforms (community‐based events [CBE] for pregnant women, CBE for well‐children and well‐child visits at facilities) to expand preventive services. Scaling these community‐based services requires addressing current challenges in the health system (including human resource and logistic gaps) and investment in demand creation for these services.

## Graphical abstract

This study uses a scenario‐based feasibility testing approach to explore potential interventions to strengthen preventive and promotive nutrition service delivery for women and children through the primary health care system. Our findings highlight that integrating preventive and promotive nutrition services requires addressing current challenges in the health system, including human resource and logistic gaps, and investing in creating demand for preventive services.

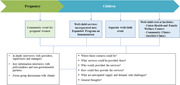

## 1 INTRODUCTION

Nutrition has become central to the development agenda with 12 of the 17 Sustainable Development Goals being directly or indirectly linked to improving it (Grosso et al., 2020). Globally, large strides have been made to address undernutrition in the past decades, yet maternal and child nutrition remains a significant public health concern, particularly in low‐ and middle countries (Black et al., 2013; Victora et al., 2021). In Bangladesh, stunting among children under 5 years declined from 60% to 31% between 1997 and 2017, wasting from 21% to 8% and underweight from 52% to 22% (NIPORT, 1997, 2020). Much of these changes in nutrition are explained by nutrition‐sensitive improvements such as increases in income, education and access to family planning, but coverage of nutrition‐specific interventions remained low (Nisbett et al., 2017). Less than half of women (47%) attended at least four antenatal care (ANC) visits, 49% had institutional delivery (NIPORT, 2020) and 35% received child growth monitoring (Nguyen, Khuong, et al., 2021).

Community‐based nutrition interventions have been found to improve maternal and child nutrition status in low‐ and middle countries (Majamanda et al., 2014) and have long been a policy focus for the Government of Bangladesh. The first community‐based nutrition interventions were implemented between 1996 and 2011 (Saha et al., 2015) called the Bangladesh Integrated Nutrition Project and later the National Nutrition Program. These programmes covered only 110 Upazilas (subdistricts) with negligible involvement of primary health care frontline workers in service delivery. A large‐scale nutrition initiative, the National Nutrition Services, began in 2011 (NNS OP, 2011, 2017), enabling the provision of mainstreamed nutrition interventions through the existing health system structure, the Bangladesh Essential Services Package. The service delivery platforms spanning health facility and community levels are mainly focused on curative services, outreach at satellite clinics, Expanded Program on Immunization (EPI) and ANC (Government of Bangladesh, 2016).

Delivery platforms such as primary health curative care facilities are less likely to invest in preventive outreach programmes (Saha et al., 2015). For example, in rural Bangladesh, growth monitoring and promotion (GMP) is integrated into facility‐based curative care, bearing several challenges related to inadequate coordination, training, supervision, logistics and supplies, hindering the implementation of GMP (Billah et al., 2017). In addition, only a subset of children is reached by services provided during sick child visits, for whom preventive services are a lower priority than curative services. Although nutrition assessment and counselling are a key component of Integrated Management of Childhood Illness, these are not prioritized (Saha et al., 2015) as there are no dedicated frontline nutrition workers (NNS OP, 2011). For pregnant women, nutrition interventions are mainly delivered during facility‐based ANC, which is a preventive platform. However, other challenges are present including low coverage of at least four ANC (NIPORT, 2020), suboptimal ANC quality (Nguyen, Khuong, et al., 2021) and persistent inequalities in the accessibility of quality care (Anwar et al., 2015; Hajizadeh et al., 2014). The frontline workers in the public health system are designated to provide nutrition services, but there are missed opportunities to prioritize and raise awareness of the importance of nutrition with families and communities since the community‐based services are primarily focused on family planning and routine immunization (Bangladesh, 2011).

To improve service coverage and quality, there is a need to strengthen nutrition services by optimally utilizing contact points, while integrating key nutrition interventions through new service delivery points. Previous assessments documented nutrition service delivery gaps (Billah et al., 2017; Saha et al., 2015), yet limited attention has been paid to the feasibility of reshaping service delivery to close gaps. Our study assesses the feasibility of strengthening and reshaping existing service delivery platforms at the community level to provide preventive nutrition services to pregnant women and young children, focusing on two key research questions: (1) What is the perceived feasibility of reshaping existing platforms or introducing new platforms to deliver preventive nutrition services? and (2) What are the barriers and facilitators to implementing these interventions?

## 2 METHODS

### 2.1 Study setting

**Figure 1:**
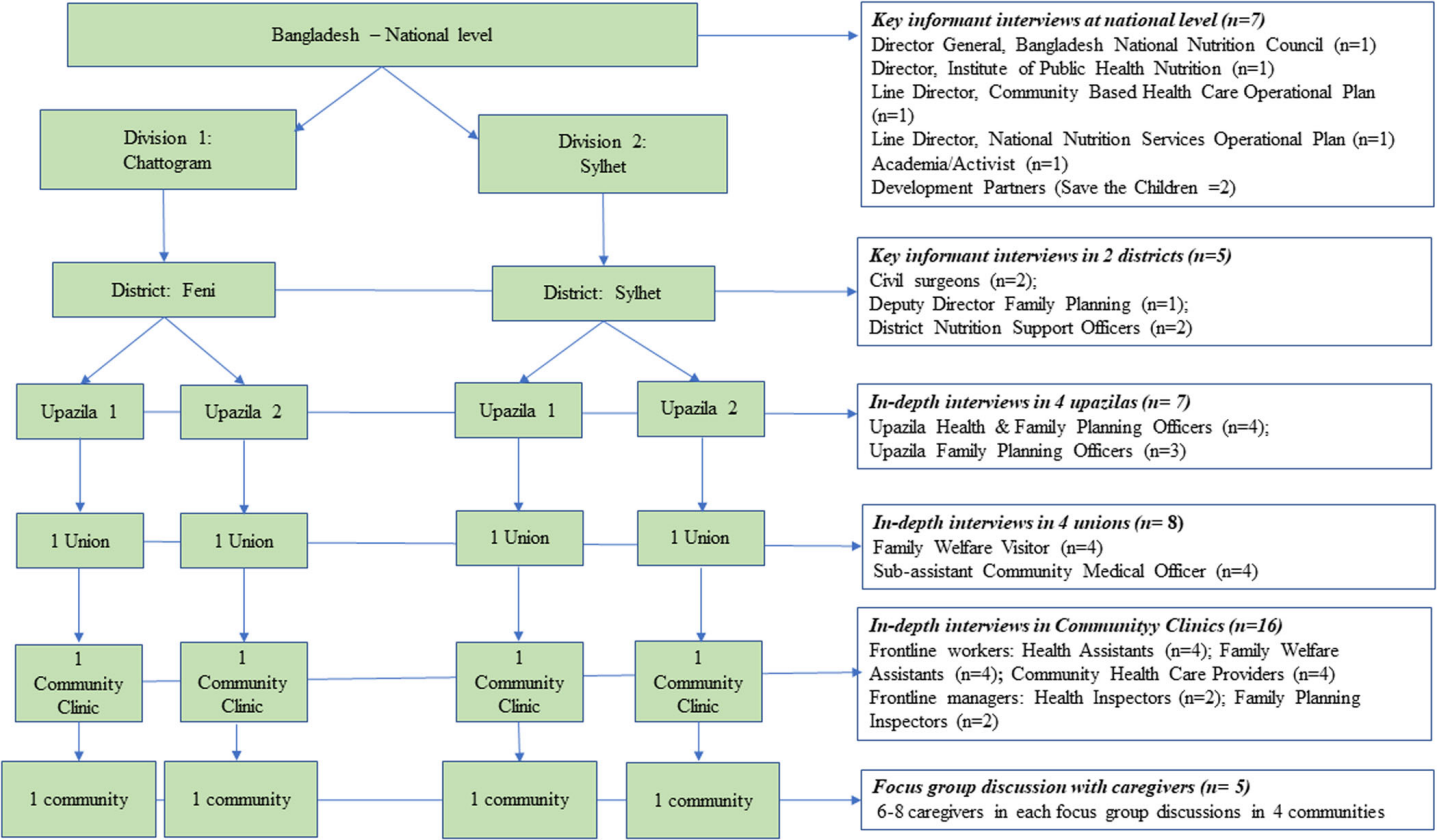
Sampling frame and sample size

This qualitative study took place in two divisions, Chattogram and Sylhet. These divisions have been prioritized by the government to strengthen the core management systems and delivery of essential health, nutrition and population services. Two districts namely Feni (Chattogram division, South‐East of Bangladesh) and Sylhet (Sylhet divisions, North‐East of Bangladesh) were randomly selected from the list of districts where the government had already completed the Competency‐Based Training on nutrition for the health system front‐line workers and front‐line supervisors. These districts have four levels of health facilities providing maternal and child health services: (1) District facilities (district hospitals and medical college hospitals), (2) Subdistrict facilities (Upazila health complexes, and other hospitals), (3) Union facilities, and (4) Community clinics. In each district, we selected two Upazilas (subdistricts), yielding a total of four Upazilas. From each Upazila, we randomly chose one union (a total of four unions), and from that union, we randomly selected one community clinic (a total of four community clinics) through a manual lottery. Details of the sampling frame are presented in Figure 1.

At the primary health care level, the Essential Services Packages for pregnant women, recently delivered women, lactating mothers and children under 5 years of age are being provided by an extensive workforce of health care providers using established facility and community‐based platforms: domiciliary visits, courtyard sessions, EPI outreach sessions and satellite clinics (primarily for ANC with some integration of EPI services). EPI outreach sessions take place following an Upazila micro‐plan at designated locations which can be an open space, courtyard of a private house, a school compound, a clubhouse or even a shop (EPI, 2018).

### 2.2 Participants

We conducted in‐depth interviews with service providers, supervisors (i.e., Health Inspectors and Family Planning Inspectors) and managers (*n *= 31, including 7 at the Upazila level, 8 at the union level and 16 at the community level) (Figure 1). We also conducted key informant interviews with policymakers at the national level (*n *= 7, including representatives of the public sector, civil society and development partners) and district level (*n *= 5, including one Deputy Director of Family Planning, two civil surgeons, and two nongovernment partners). We conducted five focus group discussions with pregnant women and mothers of children <2 years (each with six to eight women).

### 2.3 Data collection

**Figure 2:**
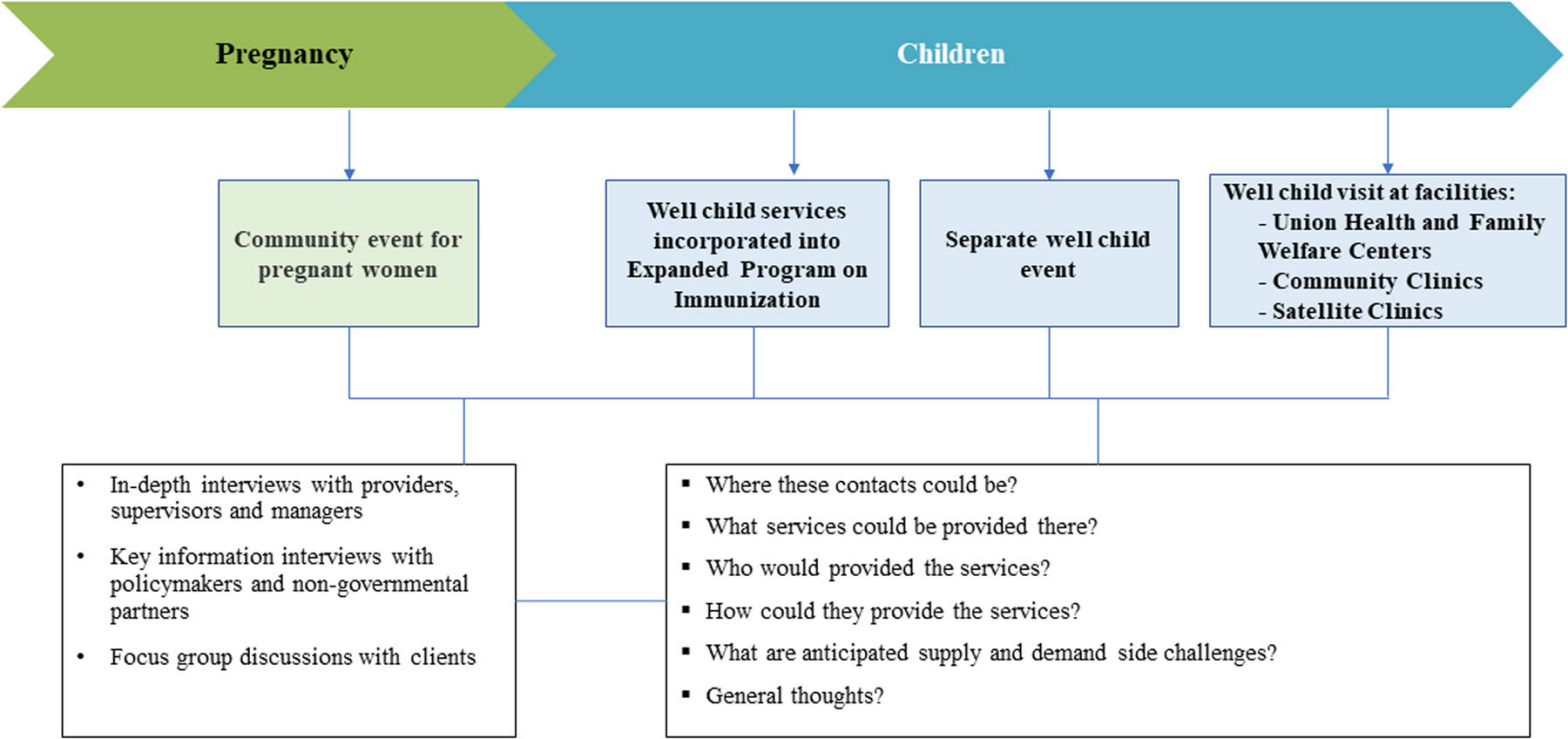
Data collection framework

We developed scenario‐based in‐depth interview guidelines for each participant category based on four potential platforms to reach beneficiaries, focusing on preventive rather than curative care (Table S1). These included one platform to reach pregnant women, and three to reach well‐children under the age of 5 years (Figure 2). The scenarios were developed by the researchers from evaluation and programme teams, in consultation with government stakeholders, based on reviewing the literature on current policy and programmes to deliver nutrition‐related services for pregnant, lactating women and children. Team discussions were used to conceptualize how each of these platforms would be defined and presented to respondents, and to frame feasibility‐related questions on where these contacts could be, what services could be provided there, who would provide them and how, and anticipated supply‐ and demand‐side challenges.

#### 2.3.1 Community event for pregnant women

This was defined as a counselling‐focused event held in the community for pregnant women, and open to family members. The discussion could centre around topics such as nutrition and care during pregnancy. Health care providers could be present to answer client questions, discuss challenges and provide counselling messages. Counselling would be group‐based and not one‐on‐one.

#### 2.3.2 Well‐child services for children

Well‐child services were presented to respondents as preventive nutrition services delivered to children either at an EPI session, a separate community event or facilities, where they would be brought for routine growth monitoring and counselling, and not when they are sick. These services would enable caregivers to receive age‐specific advice and counselling messages on feeding and caring for their children. Messages or referrals would also be tailored to the child's nutritional status, which would be assessed through services such as length/height, weight and/or mid‐upper arm circumference measurement. Potential platforms for young children could either be fixed‐day/fixed‐service (such as integrating services into EPI or holding a separate community event) or a routine service available at facilities (such as a well‐child visit protocol at facilities).

Providers, managers and policymakers were asked what kind of nutrition services could be provided through these platforms, how feasible these are, which providers would be responsible for delivering services, how to implement the events and key considerations. Beneficiaries specifically were asked whether these services would be useful to them, why they would or would not utilize them and what kind of challenges may arise.

All interview guidelines were pretested in the Bhaluka district of the Mymensingh division by experienced Research Investigators and Research Assistants to check on the flow, contents and consistency of the questions, and to contextualize the guidelines in real settings. All feedback from pretesting interviews was discussed and incorporated into guidelines. Data were collected by a team of well‐trained qualitative researchers from icddr,b. Interviews were conducted in Bengali and recorded in their entirety with consent from the respondents. All focus group discussions were conducted by at least two researchers, including one facilitator and one notetaker.

### 2.4 Data analysis

Data analysis took place using the framework approach (Smith & Firth, 2011), providing a systematic structure to manage, analyse and identify themes (Ritchie & Spencer, 1994). Recorded interviews were transcribed verbatim in Bengali. Field notes and interviewers' observations were incorporated into the transcripts. Transcribed data from the early interviews were compared to assess how similar issues were discussed by different types of interviews and to identify gaps in data exploration which could be investigated further during subsequent interviews. Before working with raw data, a set of a priori codes were identified based on interview guidelines and study objectives, allowing for emergent codes during the analysis (Table S2). The final code list was developed when all interviews were coded and condensed. Transcripts were analysed by identifying emerging themes and subthemes and highlighting common ideas and recurrent themes. Key issues, concepts and themes were based on the objectives of the study. Finally, data were systematically indexed and coded, synthesized, and interpreted. Results on the same issues from different types of respondents and areas were compared to strengthen the validity of the findings. To ensure quality, multiple researchers coded the same transcripts and at least two researchers coded each cadre of interviews, with regular discussions to resolve divergent results.

### 2.5 Ethical clearance

Written consent was obtained from pregnant women, lactating mothers and health care providers before their participation in the study and for recording interviews. The research protocol received ethical clearance from the Institutional Review Board at the International Food Policy Research Institute and icddr,b.

## 3 RESULTS

### 3.1 Characteristics of study participants

Among participants involved in key informant and in‐depth interviews, 55% were male and 59% were aged 25−44 years. Nearly half had a postgraduate degree while 27% completed higher secondary education. Considering their professional experience, 45% had 1−9 years of work experience and 32% had 20−29 years of experience. All participants in the focus group discussions were female in the young age group (85% aged 20−29 years). Around 80% of them completed primary education and most of them are homemakers.

### 3.2 Perception of the feasibility of community events for pregnant women

Almost all health service providers (*n* = 16), a few health managers (*n* = 2) and national‐level policymakers (*n* = 3) opined that organizing a counselling event for pregnant women on basic ANC components is feasible (Table 1). While some health service providers suggested that this event can be arranged in a certain place (such as a house, school or the community clinic), some other health managers and national‐level policymakers further added that such events can be arranged at the EPI sessions and community clinic instead of finding a new place.

We have satellite clinics to reach the pregnant mothers which are organized by Family Welfare Assistant. The Family Welfare Visitors visit the outreach centers twice a week and provide ANC, postnatal care, childcare and various family planning methods. So, there is no need to arrange a separate event as these are available in satellite clinics. (IDI‐30, UFPO)

**Table 1:** Perception on the feasibility of community event for pregnant women

	Providers (in‐depth interview)	Managers (in‐depth interview)	Policymakers (key informant interview)	Mothers (focus group discussions)
Feasibility	Providers feel that a community event for pregnant women is feasible and provided ideas on how it can be done Providers expressed willingness to support these events if they took place in the community	Managers feel that organizing these events is feasible, but some believe that a separate event is not necessary ANCs are already provided by skilled Family Welfare Visitors from satellite clinics at the community level These existing satellites can be strengthened by ensuring an adequate supply of medicines and motivating providers	Policymakers had mixed views about a separate community‐based event for pregnant women Some noted a separate event can be arranged in the community Other noted that a separate event is not feasible because of: o shortage of health staff o beneficiaries' lack of awareness o family restrictions	Beneficiaries were interested in a separate community event for pregnant women but noted that time and place should be convenient
How to organize the events	Doctors and paramedics should be present to provide services on these platforms Events can take place in a home, or at a school or community clinic Events should take place once a month, preferably in the morning Respondents may be referring to a ‘fixed‐day, fixed‐service' solution Local leaders can help providers find a suitable place for the event A qualified doctor is needed to provide services The provider should be female	These should be arranged without expecting the support of the Deputy Director Family Planning staff A dedicated workforce providing nutrition services is needed to conduct these events Events for pregnant mothers can be held once a month, or once every 3 months The event can be arranged at an EPI centre or community clinic Basic ANC components can be provided The session should be held in the morning on weekday	Satellite clinics and outreach centres can be utilized instead of arranging a separate event	The event should take place within the community or nearby area The events should take place in the mornings, i.e., between 10 AM−12 PM The event duration should not be too long (<1 h)
How to create demands for the event	Women can be incentivized to attend Women and their families can be mobilized by local leaders Health Assistants and Family Welfare Assistants can encourage mothers and family members to attend these events during routine home visits Nominating one or two mothers to encourage others could also boost participation	Beneficiaries may need incentives to attend	Community front line health workers can generate awareness and create demand through interpersonal communication	Arranging female health care providers can motivate community pregnant women to seek care The event should be arranged in a convenient place and time
Challenges	Beneficiaries are not motivated to come	Lack of qualified staff Lack of provider motivation Lack of logistics Lack of funding Beneficiaries are not motivated to come Lack of suitable location Incentives needed to encourage mothers to come	Beneficiaries are not motivated to come Mothers/caregivers are not allowed to attend this event by other family members Mothers/caregivers and family members do not know about nutrition, malnutrition, moderate nutrition	Mothers showed unwillingness to attend Sometimes husbands and mother‐in‐laws restrict the mothers to take part in such events

Abbreviations: ANC, antenatal care; EPI, Expanded Program on Immunization.

For the frequency and timing, most respondents suggested that events should be held once a month, but some suggested once every 3 months. Mornings were identified as the convenient time for women as they have a huge workload at noon. A health manager further explained that such events must be arranged on a workday.

It would be good if it is arranged once a month, I wish a doctor could join me to provide services. (IDI‐01, CHCP)

One health manager suggested the existing providers will be able to carry out the tasks of a separate event for pregnant women, but others (*n* = 2) cautioned though, that in such an event the provider would be Health Assistant and Family Welfare Assistant who needs to be trained first.

This separate arrangement can be made with the help of Health Assistant because he/she has a list of pregnant mothers. Mothers who have been recently registered within last 15 to 30 days can be invited to a certain place in the community for this service. (IDI‐06, HA)

In spite of a positive perception of the feasibility of a community event for pregnant women, health service providers, health managers and policymakers raised several challenges including shortage of health staff, limited training, lack of motivation in both service providers and beneficiaries, lack of funding to arrange a new event and provide incentives for mothers, and challenges finding a suitable place to hold this event.

Community Health Care Providers (CHCP) in community clinics are not skilled health workers … S/He may consider a pregnant woman to be healthy while she may have oedema, and will not recognize undernutrition. It would be good if a technical person or medical officer could be set here. (KII‐41, Civil Surgeon)

The challenge is CHCPs are not experts on counselling though trained and that's why they cannot provide proper counselling. A CHCP does not even have the concept of nutrition‐related counselling. They think nutrition is related to wealth. They do not know that home‐based food can sufficiently provide vitamins. There is a huge gap in knowledge. (KII‐40, Civil Surgeon)

Actually, we do not have time or all types of counselling … We are always tensed to fulfil targets of long‐term and short‐term family planning methods … we talk about nutrition when there is spare time. (IDI‐10, FWA)

The situation I dislike most is that when I work on one thing, then another three or four assignments are imposed upon me. Therefore, I cannot finish either of them properly. If I do any work and if I cannot maintain its quality, what is the point of working? (IDI‐06, HA)

### 3.3 Perception of the feasibility of well‐child services

Well‐children are not usually brought to community clinics for preventive nutrition services. Mothers mainly bring children to the facilities when they are sick, and only a few ask for weight measurement or ask for feeding counselling as a secondary/opportunistic reason. Well‐children are brought to facilities and other service contact points during specific service provision days, such as scheduled EPI outreach sessions or vitamin A and deworming campaign events. Overall, providers, their supervisors, policymakers and beneficiaries had mixed views on the need to provide preventive nutrition services to children.

If we continuously monitor the weight and height of a child, it will definitely prevent the child from getting malnourished. (IDI‐04)

Well children need not visit any sort of health facilities including community clinics. (IDI‐22)

No need to measure the height and weight of all children, we will measure the children whom we suspect to be malnourished. (IDI‐22)

Three potential platforms with a preventive focus as opposed to a curative focus were proposed for children including (1) Well‐child services incorporated into EPI, (2) a separate well‐child event held in the community, and (3) a well‐child visit at facilities such as Community Clinics, Union Health and Family Welfare Centers and Satellite Clinics. Satellite Clinics, managed by the health system, are generally hosted in a villager's house voluntarily provided by the owner.

### 3.4 Well‐child services incorporated into EPI

Mixed reactions were found regarding the feasibility of providing preventive nutrition services (i.e., measurement and counselling) in EPI sessions, ranging from enthusiastic positive to strong negative (Table 2). Preventive nutrition service in EPI was seen as possible only with a dedicated additional workforce to take the measurements and provide counselling services. For the timing, all preventive nutrition services for children must take place before they are immunized, and children who have received all their immunizations are less likely to be reached through this platform.

Yes it can be done. (Beneficiaries) will have to be seated and given time (to relax) before immunization, counselling can be done during that time. (IDI‐01, CHCP)

**Table 2:** Perception on the feasibility of well‐child services incorporated into EPI

	Providers (in‐depth interview)	Managers (in‐depth interview)	Policymakers (key informant interview)	Mothers (focus group discussions)
Feasibility	Providers shared mixed opinions on the feasibility of introducing nutrition services into EPI sessions Most providers felt that height and weight measurement during EPI sessions is possible Other providers felt that this is not feasible because o Currently, they already struggle to immunize all children at EPI sessions o Weight machines and height measuring boards are not available at EPI centres (other than Community Clinics)	Managers also had mixed reactions Most supported the idea of measuring children's height and weight during EPI sessions Some felt that growth monitoring could not be integrated into EPI activities because of o Lack of equipment o Lack of motivation among providers o Mothers rushed to bring children home after the EPI session	National‐level policymakers unanimously acknowledged the need for regular GMP and well‐child visits Many considered EPI centres as a potential platform to reach well‐children for preventive nutrition services Some felt that integrating GMP into EPI is not feasible because of the chaotic and noisy environment	Beneficiaries shared concerns about accessing preventive nutrition services for their children during EPI sessions All mothers do not bring their children to the EPI centre at the same time Once the injection is given, children will start crying Children who have received all their immunizations can no longer be reached through this platform
How to organize the events	A dedicated workforce is needed—one provider should take the measurements while another provides counselling Community Clinics are a feasible option, as necessary equipment is available there. Community Health Care Providers can provide counselling after height and weight are measured GMP activities should take place before immunization—afterwards, the babies will be upset, and caregivers will not be attentive	Nutrition services must take place before immunization	At least two staff are required—one for taking measurements, and one for counselling Nutrition services must take place before immunization Collaborations between the government and NGOs to provide technical support	Any services must be given before immunization
How to create demands for the event	Mothers/caregivers should be given refreshments or money as an incentive	Need to arrange courtyard meeting with the mothers or caregivers of the children to encourage them to attend	The mass media such as radio or television should be used to inform people about the service The Community Group and Community support group should be involved to increase service utilization. Caregivers/mothers should be informed through Adolescent clubs and school scout groups	Mothers should be informed about the date of this event well ahead Before the event, the Miking from Mosque (the announcement with a loudspeaker) should be done/arranged
Challenges	Logistics are not available at the EPI centre Providers already struggle to immunize children who come to the EPI sessions, and anthropometric measurement will be an added burden	Logistics are not available	Some mothers/caregivers may not be able to come on that particular date Logistics are not available there	Children cry for long time after the immunization Caregivers/mothers are in a hurry after the immunization for going back home to cook food for family Noisy environment of EPI session

Abbreviations: EPI, Expanded Program on Immunization; GMP, growth monitoring and promotion.

Several challenges are raised by providers and managers. Providers mentioned that they already struggled to immunize all children present at the EPI sessions; thus, adding nutrition services within this busy schedule would not be feasible and would add to their burden. Policymakers, however, mentioned the Multipurpose Health Volunteers, a recently established pay‐for‐performance volunteer, could have a role to address some of these challenges. A few national‐level policymakers cautioned that integrating GMP into the EPI platform would not be possible considering its chaotic and noisy environment. Weight machines, length/height measuring boards and tapes to measure mid‐upper arms circumferences are not readily available at EPI outreach sessions, unless arranged at Community Clinics, and would need to be transported through EPI logistics channels to outreach sessions, which will be difficult.

In my opinion, there are two places where this programme can be arranged: one is the EPI centres and another one is the Community Clinics. Instead of thinking of a new place, it would be good to focus on EPI centres. But it would be difficult to carry the logistics to EPI centres and these stuff are heavy. (IDI‐25)

Logistics will be needed: height machine, blood pressure machine … if logistics from community clinics are shared, community clinic services will be hampered. (IDI‐01, CHCP)

There will be problems: children cry too much on EPI days … no one will listen with patience to counseling during EPI sessions … seating arrangements will not be possible. (IDI‐04, CHCP)

Mothers hurry even when they come for immunisation. Mothers always say that they came while cooking, and each one requests to prioritise her child first! (IDI‐09, FWA)

Most mothers disliked this event idea. Mothers reasoned that all mothers do not come to EPI centres together at one time and after immunization children start crying.

Children get fever after immunisation. Many of them cry. On EPI days, it will not be possible to spare extra time for children. (FGD‐36)

### 3.5 Separate well‐child event

In contrast with integrating well‐child service within EPI, the separate well‐child event was thought to be feasible and desirable by most respondents (Table 3). Most of them noted that the event can take place once a month, and some suggested once a week, on a designated day. It was suggested that the event take place at EPI sessions, Community Clinics or union‐level facilities, but not at home. Most providers and beneficiaries considered mornings as the most convenient time. Some Community Health Care Providers, however, explained that morning sessions will not be feasible for them to attend as Community Clinics cannot be closed for such special events, and further pointed out that afternoons are beyond their working hours. Notably, providers saw familial opposition as a demand‐side challenge, but beneficiaries did not bring this up as a barrier to utilizing services.

(These events) should be Community Clinic‐oriented—I told you, counselling should be done well so that beneficiaries come to Community Clinics. (IDI‐39, UFPO)

Time should be managed (on arranging these events): the government and authority should ponder on it … it will be good if incentives can be given for that day to the health providers present on that day. (IDI‐24, HI)

**Table 3:** Perception on the feasibility of the separate well‐child event

	Providers (in‐depth interview)	Managers (in‐depth interview)	Policymakers (key informant interview)	Mothers (focus group discussions)
Feasibility	Providers felt that organizing a separate well‐child event is feasible Providers felt that mothers may be interested in this platform, but may face opposition from their families	Managers are confident about the feasibility of arranging the event	Arranging a separate event for well‐child is feasible if there is dedicated staff for this	Beneficiaries were also positive and shared convenient times and venues for a nutrition‐focused community event In contrast to provider and manager responses, beneficiaries did not mention family opposition as a barrier to attending events
How to organize the events	Human resources: Involvement of Health Assistants, Family Welfare Assistants and Community Health Care Providers. NGO support is needed Most providers suggested having the event once a month, a few suggested weekly These can be held at EPI centres on non‐EPI days or at Community clinics. It should not be held in a home or at a satellite clinic Afternoons are the preferred time by Community Health Care Providers (Community clinics cannot be left unattended)	Incentivize existing staff instead of hiring additional workers Should be arranged once a month Could be held at union‐level health facilities, where Subassistant Community Medical Officers, Family Welfare Visitors and pharmacists are present Local leaders can help procure funds to run the event	This event can be arranged by involving local elite people and local government representatives for mobilizing the community It can be arranged once a month in a selected place like a community clinic, satellite clinic, etc.	They preferred late morning when they have free time Suggested Community Clinics and schools as possible venues
How to create demands for the event	Events taking place in the morning are most convenient for mothers	Community sensitization and mobilization by Health Assistants, Family Welfare Assistants, local leaders, community groups and community support groups. Announcement through public address system at a local mosque Incentivizing mothers to participate using gifts or snacks	The community people should be mobilized with the help of local government and local leaders Multipurpose health volunteers can visit households to inform mothers/caregivers about the place and time of the event	This event should be arranged in the afternoon around 4 PM. Qualified doctors from outside (not the local health worker) should come and attend different sessions
Challenges		The community‐level health workers are overburdened Community health workers are not given transport allowance	This may not be sustainable after the end of the project Lack of human resources and training	Caregivers/mothers have household work in the morning Not possible to attend the meeting without permission from husband and mother‐in‐law

Abbreviation: EPI, Expanded Program on Immunization.

Almost all service providers and higher officials considered this to be a feasible service delivery point and offered some suggestions on how to generate demand for this event, including community sensitization and mobilization by providers or local leaders or Community Groups (members of Community Clinics management committee), announcement through public address system at a local mosque, and incentivizing mothers to participate using gifts or snacks.

It will be good if a doctor is present as mothers always see us. If we say an external doctor is coming, they will be motivated (to attend the event). (IDI‐01, CHCP)

If we inform or motivate the previous day, mothers will come … all will not come, some will be left out, but more than previous will come. (IDI‐10, FWA)

All (mothers) can be informed when mothers come for children's vaccination. Also, microphone from the masjid can be used for informing. Mothers will give it importance if (information is announced) through miking (loud speaker). (FGD‐32)

### 3.6 Well‐child visits at facilities

Well‐child visits at facilities such as Community Clinics, Satellite Clinics, and Union Health and Family Welfare Centers appear to be more feasible as they have the required workforce and logistics and can be used to create a preventive service that can also reach children who have completed their EPI (Table 4). Health care providers like Community Health Care providers, Health Assistants and Family Welfare assistants can work together to ensure both essential health and nutritional services. For measuring the weight and height of children, mothers will be invited to Community Clinics on a fixed date of a month. To create demand, it was suggested that mothers and other family members can be informed by members of the Community Group, Community Support Group, union Parishad members, mothers who visit the Community Clinics for treatment, adolescent clubs, and school scouts, religious leaders and local elites/leaders. Several challenges related to logistics and manpower are mentioned, including lack of anthropometric equipment, shortage of health workers, high workload and lack of knowledge and motivation for busy frontline workers.

If a separate event has to be arranged, it will be good to arrange it in Community Clinics instead of other places. But non‐EPI days will have to be selected (for such events). (IDI‐04, CHCP)

Logistics will be needed: height machine, blood pressure machine … if logistics from community clinics are shared, community clinic (services) will be hampered. (IDI‐01, CHCP)

It will be good if (event is arranged) in the community clinic. (FGD‐36)

**Table 4:** Perception of the feasibility of well‐child visits at facilities such as Union Health and Family Welfare Centers, Community Clinic, or Satellite Clinics

	Providers (in‐depth interview)	Managers (in‐depth interview)	Policymakers (key informant interview)	Mothers (focus group discussions)
Feasibility	Providers felt that community clinics are well‐equipped to launch these platforms	Arranging growth monitoring and nutrition services at Community Clinics is feasible	National‐level respondents feel that Community Clinics have an important role to play in any well‐child platform	Most beneficiaries are not as aware of the availability of child‐focused nutrition counselling and care at the facilities Most beneficiaries had not received a GMP card Many had never even heard about the cards and did not know that they should ask for them A few beneficiaries reported that their Community Health Care Providers had never discussed GMP with them
How to organize the events	Providers can work jointly to provide services Community Clinic‐based EPI sessions can be used to initiate the first visit	Health care providers like Community Health Care Providers, Health Assistants, and Family Welfare Assistants can work together to ensure the nutritional services Need to solve the issues of shortages of logistics and manpower	For measuring weight and height of children, mothers can be invited to a community clinic on a certain date of a month	This can be arranged at Community Clinics or school but not one's residence It has to be organized on a fixed date of a month
How to create demands for the event	Participation of children will need to be ensured by motivating parents and family members through counselling/demand creation Nutrition supplements may be distributed to children as an incentive to encourage them to come for well‐child visits	Interpersonal counselling by Health Assistants can be used to encourage parents to bring their children to GMP events Health education programmes should be promoted in the community to create awareness Children who have recently completed vaccinations can be reached through the Community Clinics	Demand creation is key. Mothers can be informed through: o Community Group o Community Support Group o Mothers who are coming to Community Clinics for treatment o Adolescent clubs o School scouts o Religious leaders o Local elites/leaders	Community health workers should motivate mothers, their family members, religious leaders, community support group members Community health workers should make a phone call to mothers before the event
Challenges	Shortage of health workers	Lack of motivation of health workers Lack of nutrition‐related training of the health workers	Community health workers are overburdened Lack of dedicated nutrition staff at the community level	Women have a scarcity of time Sometimes husbands/mother‐in‐law discourage them to participate in such event

Abbreviations: EPI, Expanded Program on Immunization; GMP, growth monitoring and promotion.

## 4 DISCUSSION

Using a scenario‐based feasibility testing approach, we have explored the feasibility to strengthen preventive and promotive nutrition service delivery for pregnant women and mothers of young children through the primary health care system. Among the four potential platforms identified, three (community‐based events [CBE] for pregnant women, CBE for well‐children and well‐child visits at facilities) were perceived as highly feasible, conditional on having the necessary workforce, structural readiness and budget support. In contrast, well‐child services integrated into immunization contacts emerged as low feasibility due to several challenges such as equipment portability, upset children and a fast‐moving service environment. Our study also highlights opportunities and challenges and offers recommendations to strengthen these services.

Previous government nutrition programmes, that is, the Bangladesh Integrated Nutrition Project and the National Nutrition Program could reach only 25% of the entire country (Saha et al., 2015). The proposed preventive nutrition platforms are consistent with the principle/strategy of delivering maternal and child nutrition interventions through health systems to ensure a wider reach. The CBE platforms for pregnant women and well‐child visits events are also consistent with the Maternal, Infant, and Young Child Nutrition (MIYCN) service delivery strategies laid out in the second national plan of action for nutrition (NPAN‐2) (Government of Bangladesh, 2017). Both NPAN2 and National Nutrition Service Operational Plan have taken a lifecycle approach in ensuring adequate nutrition for all Bangladeshi women in the first 1000 days, emphasizing routine nutrition counselling during pregnancy and GMP of children by strengthening preventive nutrition services as well as improving community engagement strategies to create demand for utilizing the services (Government of Bangladesh, 2017; NNS OP, 2017).

The existing Primary Health Care infrastructures reach up to the village level and are very favourable for community‐based platforms for MIYCN services (DGHS, 2015). The proposed preventive nutrition service platforms would complement the nutrition service delivered through existing service delivery platforms. Health care providers, their supervisors, managers and policymakers noted that existing platforms can be leveraged to provide nutrition services. Union Health and Family Welfare Centers can be used to deliver preventive nutrition services to both pregnant women as well as under‐5 children since these facilities already have skilled providers, logistics, supplies and infrastructure (NIPORT, 2019). *Maa Shomabesh* (Mother's Group Meeting) was mentioned by respondents as an existing similar event that could be utilized. Nevertheless, *Maa Shomabesh* is only being implemented in selected rural Upazilas indicating the low potential to be utilized at scale. Nutrition Services can be integrated into events held for women at the Community Clinics, and programmes implemented by the Union Parishad for women and children. Utilization of the proposed platforms especially in the poorer segments of the population could be linked with existing social safety‐net programmes (A. U. Ahmed et al., 2009), for example, the Ministry of Child and Women Affair's programme on conditional cash transfers to pregnant and lactating women. With the increased share of the for‐profit private sector in health care service utilization, engagement of the private sector to provide appropriate MIYCN service is necessary given the current concerns about quality, cost and equity of Maternal and Neonatal Health care in the private sector (Anwar et al., 2016; Rahman, 2022).

Role clarification for conducting the proposed preventive nutrition service delivery through fixed day fixed service events has been raised as an important concern given the workload and gaps in existing human resources. Though Bangladesh is one of the very few countries that has successfully scaled up and sustained its community‐level workforce (El Arifeen et al., 2013), the country has only 3.9 community health workers per 10,000 population (DGHS, 2015). Given the current shortage of qualified providers, inappropriate skills mix and inequity in distribution (S. M. Ahmed et al., 2011), a wider human resource demand and policy review is imperative to analyse the capacity of existing human resources for MIYCN services. A feasible mix of providers supported by relevant skill strengthening is necessary. Providing nutrition services through proposed platforms may also require additional nutrition‐focused staff, collaborating with NGO partners, and incentivizing workers (Heidkamp et al., 2020; Nguyen, Avula, et al., 2021). The Government of Bangladesh has introduced community‐level Multipurpose Health Volunteers who could be leveraged with cross‐operation plan collaboration under the leadership of National Nutrition Services to create demand and utilization of preventive nutrition service platform. However, these volunteers are operating on a pay‐for‐performance arrangement, and they receive payments from the respective Operational Plans they provide services for. In the current National Nutrition Service Operational Plans ending in 2022, no such budgetary provision has been made (NNS OP, 2017) to utilize Multipurpose Health Volunteers for MIYCN preventive services. To make them available for community‐based MIYCN services, National Nutrition Services should prioritize budgetary allocation for the purpose.

Over the years there have been consistent increases in ANC‐seeking, facility delivery and care‐seeking for childhood illness (Billah et al., 2017). The societal and cultural barriers to care‐seeking outside the home are diminishing. Increasing the awareness among both parents and health care providers as well as ensuring consistent availability of quality care is likely to improve care‐seeking preventive nutrition services (Billah et al., 2017). To generate awareness of and demand for these new services, communities should be sensitized through interpersonal counselling and courtyard meetings, and the involvement of community leaders and community groups. Other suggested strategies to improve attendance in CBE include providing free food or snack for beneficiaries, selecting convenient times and places for both providers and beneficiaries, making female health care providers available, even after clinic hours and outside the clinic, and activating and scaling up the involvement of Community Groups, Community Support Groups and Multipurpose Health Volunteers.

This study is influential for informing future implementation research on community‐based platforms to strengthen MIYCN services. The scenarios were carefully framed based on prior research and existing knowledge of the health system in Bangladesh. We acknowledge the limitation that responses to the potential platform could be aspirational or overly negative depending on the current workload and circumstances of the participants because they have not fully experienced some of the platforms. To minimize the response bias, we have detailed the hypothetical platform scenarios, given relevant examples from existing practice, and increased probing. The study did not include key informants from the private sector. Investigating their perspective on the nutrition service at health care contacts at for‐profit private facilities could complement the public service platforms.

## 5 CONCLUSION

A scenario‐based assessment efficiently identified potential platforms to bring MIYCN preventive and promotive services closer to the community in Bangladesh. The approach successfully identified the bottlenecks and pertinent system strengthening areas and enabling factors for such services to be successful. Integrating and scaling up preventive and promotive MIYCN services would require addressing current challenges in the health system, including human resource and logistic gaps, and investing in creating demand for preventive services.

## AUTHOR CONTRIBUTIONS

Phuong Hong Nguyen: Design study design and tools; conceive paper; analysis; draft manuscript; consolidate comments from all co‐authors; revised and finalize the paper. Priyanjana Pramanik: Conceive paper; coordinate fieldwork; analysis; draft manuscript. Rasmi Avula: Supported in developing interview guide; data interpretation and its implications; reviewed and edited the manuscript. Sk. Masum Billah, Tarana Ferdous, Bidhan Krishna Sarker, Musfikur Rahman: Coordinate fieldwork; data management; analysis; data interpretation; reviewed and edited the manuscript. Santhia Ireen: Reviewed study tools; data interpretation; and its implications reviewed and made a technical contribution to the manuscript. Zeba Mahmud: Data interpretation and its implications; reviewed the manuscript. Purnima Menon: Design study; overall management; supported data interpretation; reviewed and edited the manuscript. Deborah Ash: Reviewed study design and tools; draft discussion; data interpretation and its implications; reviewed and edited the manuscript. All authors read and approved the final submitted manuscript.

## CONFLICTS OF INTEREST

The authors declare no conflicts of interest.

## Supporting information

Supporting information.

## Data Availability

The data that supports the findings of this study are available in the tables/figures and in the supplementary material of this article.
