# Supplementary material for: Using scenario‐based assessments to examine the feasibility of integrating preventive nutrition services through the primary health care system in Bangladesh
Source: Matern Child Nutr. 2022 May 4;18(3):e13366. doi: 10.1111/mcn.13366 (PMC9218316; doi:10.1111/mcn.13366)
Supplement: Supplementary file 1 — Supporting information. [file MCN-18-e13366-s001.docx]

**Supplemental Table 1: Scenario-based interview guidelines**

| 1. **Key Informant Interview Guidelines**   **Respondents: National-, district- and sub-district- level officials**  Since we have platforms and cadres in the root level primary health care facilities, these can be further utilised for reaching all sick and will children, and pregnant mothers. We know that there are many beneficiaries who are still not reached. We would like to learn about your ideas on how to reach them with preventive services.   1. What do you think about the people that are **not** being reached by these existing services? 2. Well children are not [regularly] measured for growth in the Community Clinics. How can we reach children and their caregivers, and encourage caregivers to bring children to the Community Clinics when they are not sick (or caregivers do not think that they are sick)?   Now we’d like to talk to you about some of our ideas to reach well children and their caregivers. **[If the respondent brought up EPI/community-based events, then ask who/when/how these services would be provided – no need to bring them up separately. Ask about each platform separately if none of them were mentioned in the response.**   1. Well children are brought to health facilities during immunization. What if we added/introduced short group counselling sessions for caregivers during EPI sessions?    - How can we implement such sessions? Who, when, how? Challenges in demand- and supply sides? How should these challenges be addressed? How can these events be promoted, and the public sensitized? 2. Otherwise, we can organize a community event, either at the clinic or at another place in the community, where caregivers could bring their children to get their weight and height measured, and receive nutrition advice/counselling according to the children’s nutritional status?  - How can we organise and implement such events? Where, by whom, when, how, how often? Challenges in demand- and supply sides? How should these challenges be addressed? How can these events be promoted, and the public sensitized?  1. We’ve also seen that ANC visit coverage is lower than expected [add data here]. What if we organize regular meetings for pregnant women in the community, where they can come to discuss issues they face during pregnancy, and get advice/counselling from a provider?  - How can we organise and implement such events? Where, by whom, when, how, how often? Challenges in demand- and supply sides? How should these challenges be addressed? How can these events be promoted, and the public sensitized? |
| --- |
| 1. **In-depth Interview Guidelines**   **Respondent: Family Welfare Visitor and Sub-assistant Community Medical Officer**  We have thought about some ways in which regular growth monitoring services and related nutrition counselling can be given, and we would like to hear your thoughts on these.  24. One solution could be that during EPI or vaccination sessions, parents can get nutrition counselling and get their children’s height and weight measured.  (a) How can this be done? [Hint: which services, who provides, where, what support/equipment/manpower is needed] [Hint: growth monitoring + counselling, only counselling, only growth monitoring]  (b) What kind of challenges may come up in doing something like this? [Hint: time, parents/providers not interested, families won’t allow] |
| 1. **In-depth Interview Guidelines**   **Respondent: Community Health Care Provider, Health Assistant, Family Welfare Assistant, Family Planning Inspectors, Health Inspectors**  So far we have talked about to major things – services that are provided to pregnant women during visits and services that are provided to sick children during visits. However, during curative care, in many cases there is no time to provide nutrition counselling properly. Parents may also not be in a position to listen to counselling properly during their child’s treatment, or be in too much of a hurry.  1. How important do you consider regular growth monitoring (height and weight measurement) for well children, and counselling on the child’s nutrition for the parents? [If the CHCP says these are necessary]: Then, how can well children be brought to the community clinic for regular growth monitoring and nutrition counselling?  We have thought about some ways in which regular growth monitoring services and related nutrition counselling can be given, and we would like to hear your thoughts on these.  2. One solution could be that during EPI or vaccination sessions, parents can get nutrition counselling and get their children’s height and weight measured.  (a) How can this be done? [Hint: which services, who provides, where, what support/equipment/manpower is needed] [Hint: growth monitoring + counselling, only counselling, only growth monitoring]  (b) What kind of challenges may come up in doing something like this? [Hint: time, parents/providers not interested, families won’t allow]  3. Another thing that can be done is that a separate event could be organized where parents can bring their young children to getweight and height measured and get nutrition counselling. This event could take place at a specified date at time, for example, once or twice a month.  (a) How can this be done? [Hint: which services, who provides, where, what support/equipment/manpower is needed] [Hint: growth monitoring + counselling, only counselling, only growth monitoring]  (b) What kind of challenges may come up in doing something like this? [Hint: time, parents/providers not interested, families won’t allow]  4. We are thinking of two ways to get regular growth monitoring and counselling services to all children. But parents and families may not be interested in these new services. What can be done to create demand for these new services among the families and increase awareness and interest? When (what time of day) should these services take place so that people can come?  5. Other than this, we are also thinking about ways to reach pregnant women with support and services. There could be a group meeting once a month, where pregnant women can talk about issues they are facing, and get counselling from a provider.  (a) How can this be done? [Hint: which services, who provides, where, what support/equipment/manpower is needed] [Hint: growth monitoring + counselling, only counselling, only growth monitoring]  (b) What kind of challenges may come up in doing something like this? [Hint: time, parents/providers not interested, families won’t allow]  (c) How can interest in this service be created among the pregnant women and their families?  (d) When (what time of day) should this service be provided so that they can come?  6. So far we have talked about ways to reach all children and pregnant women in the area around the community clinic with nutrition services. What are the ways that people can get to know about the new services in time? In your area, how is this kind of information circulated around the community?  7. What is your work pressure like right now? If you are involved in these new services we are thinking of, how will that affect your work pressure? Considering your current workload, will it be possible? [If no:] How can this be done without increasing your work pressure? |
| 1. **Focus Group Discussion Guidelines**   **Respondents: Mothers of u-5 children and pregnant women**  We have thoughts of some ways to reach children with nutrition servies even before they fall sick. For example: regular height and weight monitoring, counselling to parents according to nutritional status etc.  15. An event can be held at the community clinic or at the area around it where everyone can bring their children for height and measurement, learn how their child is growing, and get counselling according to nutritional status. Will mothers in the community actually come to such an event if it happens? If no: Why not? [Help: children are not sick, household work, opposition from family]  16. If nutrition counselling is given for the children at the EPI centre, will that be useful for the mothers? Why or why not?  17. If an arrangement is made only for pregnant women, where they can meet with a provider once a month to discuss issues and get counselling, will the pregnant women in your community come? If not: Why not? [Help: no issues faced, household work, opposition from family]  18. [If opposition from family is mentioned]: How can we sensitize husbands and other family members so that they allow the women to come to these events and give them support? [Help: who would they listen to, sensitization by providers, mic announcements, community elders, word of mouth]  19. Where and when should these events/meetings take place so that it is convenient for you and you can attend? |

**Supplemental Table 2: Example of code list for potential platform study**

| **Code number** | **Priori code** | **Description of code** | **Final code** |
| --- | --- | --- | --- |
| - - 1. **In-depth interview** | | | |
| 6.a | Necessity measure height/ weight | Necessity of measuring height and weight of well children, and giving nutrition-related advice to caregivers | Need for measuring height and weight for providing preventive nutrition |
| 6.b | Bring well child to Community Clinics | How to bring well child to Community Clinics for weight and height measurement, and nutrition counseling? | Strategy to bring well-child to Community Clinics |
| 6.c | Feasibility EPI platform | Feasibility and opinion of using EPI as a platform for weight and height measurement of well children, and nutritional advice to their caregivers. | Exploring the possibility of providing nutrition services at EPI |
| 6.d | Support CHCP | Possible support from Community Health Care Providers on this occasion | The ways how Community Health Care Providers can contribute to this event |
| 6.e | challenges | Possible challenges to initiate this event | The major challenges related to this |
| 6.f | Feasibility separate arrangement | Feasibility and opinion of a separate arrangement/event where parents will bring their children to measure height and weight, and get nutrition counseling accordingly | Finding alternative way to reach well-child other than EPI platform |
| 6.g | Support CHCP | Possible support from Community Health Care Providers on this occasion | The ways how Community Health Care Providers can contribute to this event |
| 6.h | Challenges | Possible challenges to initiate this event | The major challenges this idea could pose |
| 6.i | Encourage parents | How to encourage parents and other family members to bring their children to these events? | Community mobilization to bring well-child to this event |
| 6.j | Convenient date/time | Convenient date and time for parents/caregivers for attending such events | Exploring the appropriate date and time to attend this event |
| 6.k | Feasibility separate arrangement | Feasibility and opinion on a separate arrangement/event (i.e. group meeting) for pregnant women to attend and discuss about their problems among themselves and receive services from provider | Feasibility of arranging a separate event for pregnant women in the community |
| 6.l | Support CHCP | Possible support from Community Health Care Providers on this occasion | Feasibility of engaging Community Health Care Providers in this event |
| 6.m | Challenges | Possible challenges to initiate this event | The major challenges this idea could pose |
| 6.n | Encourage pregnant women | how to encourage pregnant women and family members to attend such meetings | Community mobilization for pregnant women and family members to attend this event |
| 6.o | Convenient date/time | Convenient date and time for pregnant women for attending such events | Exploring the appropriate date and time to attend this event |
| 6.p | Ways circulate | Ways to circulate about the new events/arrangements in the area | Ways to reach people with the news of this new event |
| 6.q | Workload | Current workload Community Health Care Providers | Current workload of Community Health Care Providers |
| 6.r | Feasibility workload | Feasibility of attending/supporting the new events/arrangements without adding to their current workload | Engaging the existing health workers without adding to their work pressure |
| 6.s | Means attend workload | Means to attend/support the new events/arrangements without adding to their current workload as Community Health Care Providers | How health workers can help considering their workload |
| - - 1. **Focus group discussion** | | | |
| 15.a | Mothers come to Community Clinics | In Community Clinics or adjacent to Community Clinics, if an event arranged where guardian may bring their children for measuring height and weight and get counselling depending on it. Whether mothers will come there or not? | Exploring the willingness of mothers/caregivers to bring their well-child to nearby Community Clinics |
| 15.b | Reasons not come | Reasons for not coming | The possible reasons for not attending this event |
| 16.a | Convenient mothers EPI | Will it be convenient to mothers if they take their children to EPI centre for immunization and then for measuring height-weight and nutrition related counselling? | Understanding the convenience of mothers to EPI for both immunization and nutrition counselling |
| 16.b | Why convenient | Why it will be convenient? | Reasons for the convenience of mothers |
| 16.c | Why not | Why it will not be convenient? | Reasons for not convenience of mothers |
| 17.a | mothers attend event | If any event is arranged once in a month for the pregnant women in a certain place within the community where mothers will get ANC and counselling, will mothers attend such event? | Exploring the willingness of mothers to attend an event to receive ANC |
| 17.b | Why not | If not, why | Reasons behind non-participation |
| 18 | Make husband/ family interested | How to make husband and family members interested so that they allow mothers to attend such event at Community Clinics or other places (if there is any barriers from family) | Understanding the barriers and facilitating factors |
| 19.a | Convenient place | Convenient place for mothers to attend | Exploring convenient place for the event |
| 19.b | Convenient time | Convenient time of the day for mothers to attend | Exploring convenient time for the event |
